# P107: Moving from standard alcoholic hand rub dispensers to a wireless LAN based system with continuous monitoring: evaluation after one year

**DOI:** 10.1186/2047-2994-2-S1-P107

**Published:** 2013-06-20

**Authors:** B Batzer, S Scheithauer, C Pino Molina, A Widmer

**Affiliations:** 1 Infection Control, Unispital Basel, Basel, Switzerland; 2 Infection Control & Infectious Diseases, University Hospital Aachen; RWTH Aachen, Aachen, Germany; 3 Hematology, Unispital Basel, Basel, Switzerland

## Introduction

Improving hand hygiene compliance is a cornerstone of any infection control program. Manual observation temporarily improves adherence, but is time-consuming and rarely feasible long term. Electronic counting dispensers allowing real time assessment and feedback of hand hygiene events (HHE) are an effective tool for monitoring compliance 24/7/365.

## Objectives

The aim of the study was to evaluate utility and acceptance, and to identify possible shortcomings of the Ingo-man Weco (Ophardt Hygienetechnik, Issum; Germany).

## Methods

In January 2012 all dispensers (N=63) at a 13 bed hematology ward with approximately 100 transplantations/year were exchanged by Ingo-man Weco. The energy for the data transfer derived from pulling the lever of the dispenser, no battery is necessary or power lines. A built-in wireless recording equipment sending the information to a server. HHE event is defined as two activities maximum 2 seconds apart. All HHEs were continuously recorded from 03-09/12 and could be analyzed dispenser-, day-, shift-, localization-specifically. At the hematology ward about 280 patients referring to 3600 patient-days were cared for annually.

## Results

Overall, 3 dispenser were placed in the each patient room, 13 in the hallways, and 11 at nursing stations and others locations. The new devices were well accepted without handling problems. During the pilot phase, 13 dispensers with very low activities were identified and subsequently relocated.

During the 7-month lasting study, a total of 123’171 HHE could be documented. The majority (65’683/123’171; 53%) occurred at dispensers located at patient’s door entry. Only 8% (10276/123171) referred to dispensers located in the patient rooms.

Four (6%) dispensers went broken. Two of them were located at the laminar air flow benches, the damage was probably driven by the UV exposition.

## Conclusion

The Ingo-man Weco is an easy to install and ready to use device, requiring no battery or power installation, that offers 24 /7/365 data on hand hygiene activities. It allows improving dispensers location, and continuous monitoring.

## Disclosure of interest

None declared

